# Harnessing T Cells to Control Infections After Allogeneic Hematopoietic Stem Cell Transplantation

**DOI:** 10.3389/fimmu.2020.567531

**Published:** 2020-10-15

**Authors:** Sabrina Basso, Francesca Compagno, Paola Zelini, Giovanna Giorgiani, Stella Boghen, Elena Bergami, Jessica Bagnarino, Mariangela Siciliano, Claudia Del Fante, Mario Luppi, Marco Zecca, Patrizia Comoli

**Affiliations:** ^1^Pediatric Hematology/Oncology, Fondazione Istituto di Ricovero e Cura a Carattere Scientifico Policlinico San Matteo, Pavia, Italy; ^2^Cell Factory, Fondazione Istituto di Ricovero e Cura a Carattere Scientifico Policlinico San Matteo, Pavia, Italy; ^3^Immunohematology and Transfusion Service, Fondazione Istituto di Ricovero e Cura a Carattere Scientifico Policlinico San Matteo, Pavia, Italy; ^4^Section of Hematology, Department of Medical and Surgical Sciences, University of Modena and Reggio Emilia, Azienda Ospedaliero-Universitaria Policlinico, Modena, Italy

**Keywords:** multipathogen infection, T cell immunity, T-cell therapy, pathogen specific T cells, Allo-HSCT

## Abstract

Dramatic progress in the outcome of allogeneic hematopoietic stem cell transplantation (allo-HSCT) from alternative sources in pediatric patients has been registered over the past decade, providing a chance to cure children and adolescents in need of a transplant. Despite these advances, transplant-related mortality due to infectious complications remains a major problem, principally reflecting the inability of the depressed host immune system to limit infection replication and dissemination. In addition, development of multiple infections, a common occurrence after high-risk allo-HSCT, has important implications for overall survival. Prophylactic and preemptive pharmacotherapy is limited by toxicity and, to some extent, by lack of efficacy in breakthrough infections. T-cell reconstitution is a key requirement for effective infection control after HSCT. Consequently, T-cell immunotherapeutic strategies to boost pathogen-specific immunity may complement or represent an alternative to drug treatments. Pioneering proof of principle studies demonstrated that the administration of donor-derived T cells directed to human herpesviruses, on the basis of viral DNA monitoring, could effectively restore specific immunity and confer protection against viral infections. Since then, the field has evolved with implementation of techniques able to hasten production, allow for selection of specific cell subsets, and target multiple pathogens. This review provides a brief overview of current cellular therapeutic strategies to prevent or treat pathogen-related complications after HSCT, research carried out to increase efficacy and safety, including T-cell production for treatment of infections in patients with virus-naïve donors, results from clinical trials, and future developments to widen adoptive T-cell therapy access in the HSCT setting.

## Introduction

Dramatic progress in the outcomes of allogeneic hematopoietic stem cell transplantation (allo-HSCT) from alternative sources in pediatric patients has been registered over the past decade, providing a chance to cure the children and adolescents in need of a transplant (1–4). Despite encouraging results, infections are still important causes of morbidity and mortality in immunosuppressed patients following HSCT (5). Viral reactivations predominantly develop within the first 6 months after HSCT. Double-stranded DNA viruses contribute to substantial morbidity, with herpesviruses, adenovirus (AdV) and polyomaviruses BK (BKPyV) and JC (JCPyV) as the clinically most relevant infections (5–14). In addition, respiratory viruses and fungal infections are also associated with dismal outcome (15–18).

If the development of single opportunistic infections may have severe consequences in transplant recipients, it has been demonstrated that persistent detection of multiple DNA viruses is frequent after allogeneic HSCT, and had a dose-dependent association with increased mortality (19). Indeed, cumulative viral load AUC in the first 100 days post-HSCT was consistently and independently associated with increased risk for early and late overall mortality and non-relapse mortality (NRM). The effects on NRM do not appear to be direct, as only a small portion of patients succumbed to viral disease. Rather, viremia may cause indirect effects due to increased production of proinflammatory and immunomodulatory cytokines that contribute to the pathogenesis of HSCT complications (20, 21).

In recent years treatment of viral complications after HSCT has improved in part because of the introduction of new antivirals, and in part from the preemptive use of antiviral agents at the onset of viremia. The latter is successful thanks to the widespread use of surveillance by molecular detection methods (22, 23). Likewise, the ability to recognize invasive fungal disease while in the early stages, by means of imaging and peripheral blood antigen measurement, coupled with assessment of antifungal immune responses, allowed for prompt treatment and amelioration of outcome (24). Despite advances in prophylactic and preemptive pharmacotherapy, anti-pathogen therapeutics are limited by toxicity, in particular myelosuppression and renal injury, and to some extent by a lack of efficacy in breakthrough infections (25).

The development of infections in the post-transplant period principally reflects the inability of the absent/depressed host immune system to limit pathogen replication and dissemination; loss of T cell function is central to this effect (26–28). T-cell reconstitution is a key requirement for effective infection control following HSCT, and factors that influence the speed of T-cell recovery also impact the risk of infection in this period (27). A high degree of HLA mismatch between donor and recipient reduces the efficacy of immune surveillance due to poor epitope recognition, and increases the risk of inducing alloimmune responses, thus requiring stronger immunosuppression to prevent and treat graft-vs.-host disease. Likewise, delayed immune recovery is associated with T-cell depletion of the graft before transplantation.

Given the central role of pathogen-specific T cells in infection surveillance, immunotherapeutic strategies to accelerate reconstitution of pathogen-specific immunity and to hasten T cell recovery after HSCT represent a compelling alternative to drug treatments (14, 23, 27, 29–36). Moreover, preventive strategies may be expanded toward the use of virus-specific T cell assays to help identify patients at risk and to tailor therapeutic intervention (23, 37–40).

Here, we discuss the clinical achievements of T-cell therapy for infections, describe the impact of technical developments on clinical applicability, and give indications on future directions to broaden access.

## Cell Therapy for Infections After HSCT

### Donor Lymphocyte Infusions

The use of donor lymphocyte infusions (DLI) derived from seropositive stem cell donors is an effective salvage therapy for viral infections in HSCT recipients prior to T-cell recovery, but the risk of potentially severe acute or chronic graft-vs.-host disease (GVHD) is a concern (41). In order to reduce the risks derived from alloreactivity associated with DLI, non-specific T cells transduced with a retroviral construct containing suicide genes, to induce susceptibility to drug mediated lysis in case of development of alloreactive response, have been employed with success (42). The use of DLI modified with the iCasp9 cell-suicide system in a small cohort of children transplanted for acute leukemia demonstrated the potential advantages in terms of rapid and consistent cell removal in case of GVHD development (43).

### Pathogen-Specific T Cells: Production Protocols

An alternate strategy consists in delivering infectious antigen-specific T cells selected by cell culture or by sorting. A major breakthrough was achieved by the adoptive transfer of virus-specific cytotoxic T lymphocytes (CTL) reactivated from the peripheral blood of HSCT donors as prophylaxis/treatment against CMV disease or EBV-positive post-transplant lymphoproliferative disease in patients given T-cell depleted, HLA-disparate, unrelated HSCT (32, 33). This approach has been successful in preventing and treating CMV and EBV infectious complications after T-cell depleted haplo-HSCT, both in the pediatric and adult setting, while limiting the risk of inducing GVHD (27, 30).

Initially, protocols for production of virus-specific T cells (VSTs) were all based on complex procedures of stimulation and *in vitro* expansion, leading to a final product of polyclonal T cells with broad specificity. One of the main advantages of *ex vivo* differentiation is the ability to overcome the hurdle of obtaining substantial numbers of VSTs from donors with low-frequency memory T cells for a given antigen, and the ability to reduce alloreactivity by continuous stimulation with viral antigens. This is counterbalanced by production times, that can be as long as 3–8 weeks, limiting its usefulness in patients with urgent clinical need and running the risk of inducing cellular exhaustion. The latter does not seem to be a major obstacle, however, as donor gene-marked EBV-specific T cells cultured for 4–6 weeks were able to reconstitute T cell memory in HSCT recipients, and were detected as late as 9 years after administration in patients with viral reactivation (44). The availability of synthetic peptide pools, novel techniques, and progress in culture reagents and vessels has allowed reduction in production time, bringing it to < 2 weeks (45–47).

A valid alternative to cell culture is direct selection of pathogen-specific T cells by using viral peptide HLA class I multimers conjugated to magnetic beads (48), or stimulation with viral peptides followed by the IFN-gamma capture assay with magnetic beads (34, 49, 50). The latter has an important advantage over multimers, as it allows selection of CD4+ in addition to CD8+ virus-specific T cells, guaranteeing sustained long-term immune protection (51). Direct selection allows rapid production of VSTs, but it is generally feasible only for pathogens inducing an ample memory T cell pool, such as for CMV or EBV, and requires a leukapheretic procedure to obtain starting cellular material. In addition, it is not an option for virus-naïve subjects.

### Pathogen-Specific T Cells: Clinical Results for EBV, CMV, ADV, and Aspergillosis

Since the early clinical trials for EBV and CMV, the prophylactic, preemptive and curative use of T cell therapy for infection has expanded, due to the reported high rates of response and low toxicity (Tables 1, 2). The efficacy of virus-specific adoptive cellular therapy has been difficult to assess, due to the difficulties of running large prospective multicenter clinical trials, and heterogeneity of reported studies in study design, cell product characteristics and treated cohorts. However, prophylaxis/preemptive treatment of EBV PTLD after HSCT has shown more than 95% response rate in the 107 patients treated with cultured single VSTs (23, 33, 44, 52, 53, 85). Treatment of overt disease was successful in over 80% of the patients treated for PTLD (52, 54–56, 85) or CMV viremia or disease (32, 35, 58–62), with little toxicity almost exclusively limited to a 1–10% rate of GVHD. The rate of GVHD was generally lower in patients treated for EBV infection/disease, probably due to a prevalence of CD8+ T cells in the infused EBV-specific CTLs, compared to a larger portion of CD4+ T cells present in CMV-specific products. Directly selected cellular products employed in more recent studies have proven equally effective in reconstituting post-transplant immunity, but rates of clinical responses were slightly lower, reportedly 60% in patients with PTLD (50, 57) and 70% in patients treated for CMV (48, 49, 63, 65, 73) or ADV (34, 66–68, 86) viremia or disease. Moreover, the incidence of new onset or exacerbation of GVHD was higher at 15%, likely due to residual, potentially alloreactive, T cells in the product. Clearly, as head-to-head controlled studies with cell products obtained by culture vs. direct selection have not yet been performed, the reported efficacy and safety rates of the different strategies may be confounded by the variety of protocols and clinical settings.

**Table 1:** Published trials using single pathogen-specific T cells.

**Virus**	**Pt n.**	**Antigen**	**LTC stimulation**	**Clinical effects**	**GVHD**	**References**
***HSCT donor-derived***						
EBV	113	LCLs	*in vitro* culture	11/13 pts achieved CR, none PTLD	8/51 pts aGvHD; 13/108 cGvHD (11 limited, 2 extensive)	(33, 52)
EBV	6	LCLs	*in vitro* culture	5 pts had EBV-DNA decreased, 1 pts died of PTLD	None	(53)
EBV	14	LCLs	*in vitro* culture	10 pts achieved CR, 4 pts progressive disease	None	(54)
EBV	1	LCLs	*in vitro* culture	No response	None	(55)
EBV	4	LCLs	*in vitro* culture	3 pts achieved CR, 1 pt had decreased EBV-DNA level without PLTD	None	(23)
EBV	15	LCLs	DCs pulsed with LCL lysate; *in vitro* culture	7/8 pts achieved CR	5 pts (33%) aGVHD (1 gr. I, 3 gr. II, 1 gr. III) 2 (13%) limited cGVHD	(56)
EBV	6	Lytic and latent EBV antigens	Peptide mix stimulation; direct selection	3 pts had CR, 3 pts had no response	None	(57)
EBV	10	EBNA1	Recombinant protein or peptides; direct selection	7/10 pts achieved CR	1 grade II aGVHD	(50)
CMV	14	CMV virions	Fibroblasts infected with CMV strain; CD8 T cell cloning	All pts reconstituted CMV-specific immunity	3 grade I or II aGVHD	(32)
CMV	8	CMV lysate	PBMCs cultured in the presence of virus lysate	6 pts cleared infection after 1 or 2 doses; 1 pt NR; 1 pt NE	None	(58)
CMV	16	Inactivated CMV virions	DCs pulsed with lyophilized CMV antigen; *in vitro* culture	All pts reconstituted specific immunity; 8/16 pt did not require antivirals	1 grade I aGVHD	(59)
CMV	25	CMV lysate	PBMCs pulsed with CMV lysate; T cell colony expansion	7/25 pts developed CMV antigenemia; 5/25 pts developed CMV disease (3 CR, 2 NR)	1 grade I GVHD	(35)
CMV	9	CMV pp65 peptide	DCs pulsed with pp65-derived peptide; *in vitro* culture	6/9 pts developed CMV reactivation; no CMV disease	3 grade III GVHD (1 fatal)	(60)
CMV	7	CMV pp65 and IE1 peptides	PBMCs pulsed with CMV peptide mixes; *in vitro* culture	5/7 had increased antiviral immunity in PB	None	(61)
CMV	16	CMV pp65 peptides	PBMCs pulsed with 15-mer CMV peptide mixes; *in vitro* culture	14/16 pts cleared viremia	None	(62)
CMV	9	CMV pp65 or IE1	Peptide-HLA tetramer selection	8/9 cleared CMV infection	2 grade I or II aGVHD	(48)
CMV	18	CMV pp65 protein	PBMCs pulsed with protein; direct selection	15/18 pts had reduction or clearance of viremia	1 cGVHD	(63)
CMV	18	CMV pp65 protein or peptides	PBMCs pulsed with protein/peptides; direct selection	1/7 pts treated prophylactically reactivated 11/11 pts treated preemptively cleared CMV	5 grade I, 3 grade II- III aGVHD; 6 cGVHD	(49)
CMV	6	CMV pp65 peptides	PBMCs pulsed with peptides; direct selection	6/6 pts cleared viremia	None	(64)
CMV	2	CMV pp65 peptides	PBMCs pulsed with peptides; direct selection	2/2 pts attained CR	None	(65)
AdV	9	Type C AdV antigen	PBMCs pulsed with antigen; direct selection	5/6 evaluable pts attained viral clearance	1 aGVHD exacerbation	(34)
AdV	30	AdV hexon protein	PBMCs pulsed with antigen; direct selection	21/30 pts had clinical/virological response	1 grade I GVHD	(66)
AdV	8	AdV hexon peptides	PBMCs pulsed with peptide mix	8/8 pts cleared viremia; 1 pt subsequently reactivated due to GVHD therapy	1 grade IV GVHD	(67)
AdV	11	AdV hexon peptides	PBMCs pulsed with peptide mix; direct selection	10/11 pts cleared viremia and/or AdV disease	1 grade I, 1 grade III aGVHD; 1 ext. cGVHD	(68)
BKPyV	1	BKPyV VP1 and LT	PBMCs pulsed with Peptides; direct selection	1 pt cleared infection and had CR	None	(69)
JCPyV	1	JCPyV VP1 and LT	PBMCs pulsed with overlapping peptides; *in vitro* culture	1 pt cleared infection and had CR	None	(14)
Aspergillus f.	10	Fungal conidia	PBMCs pulsed with conidia; T cell colony expansion	9/10 pts attained CR	None	(35)
***Third-party donor-derived***						
EBV	33	LCLs	*in vitro* culture	14 pts attained EBV CR, 3 pts had PR, 16 pts no response at 6 m	None	(70)
EBV	5	LCLs	*in vitro* culture	4 pts attained EBV CR, 1 pts progressive disease	None	(71)
EBV	33	LCLs	*in vitro* culture	CR or PR was achieved in 68% of HSCT recipients. For patients who achieved CR/PR or SD after cycle 1, 1 y OS was 88.9%	1 grade I skin aGvHD	(72)
EBV	1	EBV peptides	Peptide-HLA multimer selection	CR after 9 m, recurrence then response to 2nd infusion	None	(73)
CMV	5	CMV pp65	Peptide-HLA multimers selection	4/5 pts attained viremia clearance	None	(73)
JCPyV	3	BKPyV VP1, VP2, VP3, ST and LT peptides	PBMCs pulsed with overlapping peptides; *in vitro* culture	2/3 pts cleared infection and CR (1 with sequelae)	1 IRIS	(74)

**Table 2:** Published trials using multivirus-specific T cells.

**Virus**	**Pt n.**	**Antigen**	**LTC stimulation**	**Clinical effects**	**GVHD**	**References**
***HSCT donor-derived***						
AdV, CMV and EBV	26	AdV5; CMV pp65; EBV-LCL	LCLs transduced with Ad5f35-pp65	6/6 with EBV cleared infection; 5/6 with AdV cleared infection; 10/11 CMV cleared infection and 1 pt progressed despite VSTs/pharmacotherapy	2 grade I GVHD	(75, 76)
AdV and EBV	14	AdV5; EBV-LCL	LCLs transduced with Ad5f35 vector	11 pts treated as prophylaxis remain negative; 2/3 pts with AdV cleared infection	3 grade I GVHD	(77)
CMV and EBV	3	CMV pp65; EBV IE1 and LMP2	DCs pulsed with peptides	2 pts cleared infection; 1 pt treated as prophylaxis remains negative	1 grade I GVHD	(78)
AdV, CMV and EBV	10	AdV5 Hexon and Penton; CMV IE1 e pp65; EBV LMP2 and BZLF1	DCs nucleofected with plasmids encoding for viral antigens	8/10 pt: complete virologic responses	1 skin rash due to GVHD or BKPyV infection	(79)
AdV, BKPyV, CMV, EBV and HHV6	11	AdV5 Hexon; BKPyV LT + VP1; CMV IE1 + pp65; EBV LMP2 + EBNA1 + BZLF1; HHV6 U11 + U14 + U90	Peptides pool from immunodominant antigens	3 pts treated as prophylaxis remain negative; 94% response rate (15 Cr and 2 PR) in 8 pts with 18 viral infections/reactivations	1 grade I GVHD	(80)
AdV, CMV, EBV, and VZV	10	AdV5; CMV pp65; EBV EBNA1 and LMP; VZV vaccine	Ad5f35-pp65, Ad5f35-EBNA1/LMP, commercial VZV vaccine	6 pts with CMV reactivation, only 1 receving antiviral therapy; no EBV, AdV or VZV reactivation	1 grade II GVHD 1 grade III GVHD	(81)
AdV, CMV and EBV	3	AdV5; CMV pp65; EBV-LCL	LCLs transduced with AdV5-pp65 vector	1 pt cleared infection. 2 pts treated as prophylaxis remains negative	None	(82)
CMV, AdV and EBV	7	Various source antigens	T cell culture; in 1 case, streptamer selection	2 pts with EBV attained CR; 5 pts with CMV:2 CR, 2 PR and 1 failure	1 grade I GVHD 1 grade II GVHD	(83)
***Third-party donor-derived***						
AdV, CMV and EBV	50	Ad5, CMV pp65, EBV-LCL	LCLs transduced with Ad5f35-pp65	6/9 pts with EBV attained CR or PR; 14/18 pts with AdV attained CR or PR; 17/23 pts with CMV attained CR or PR	6 grade I GVHD 2 grade II GVHD	(84)
AdV, CMV and EBV	4	Various source antigens	T cell culture	1/2 pts with EBV attained CR or PR; 1 pt with AdV cleared infection; 1 pt with CMV reactivation required specific pharmacotherapy.	None	(83)
AdV, BKPyV, CMV, EBV and HHV6	38	AdV5 Hexon; BKPyV LT + VP1; CMV IE1 + pp65; EBV LMP2 + EBNA1 + BZLF1; HHV6 U11 + U14 + U90	Peptide pools from immunodominant antigens	3/3 pts with EBV attained CR; 8/10 pts with AdV attained CR or PR; 20/21 pts with CMV attained CR or PR; 19/21 pts with BKV attained CR or PR 3/3 pts with HHV6 attained CR or PR;	2 grade I GVHD *de novo*; 4 grade I-III recurrent GVHD	(36)

Attempts at reconstituting cellular immune responses to fungal antigens, and controlling invasive aspergillosis (IA) in HSCT recipients have been also successful. Pioneer work showed the feasibility to expand T cell clones directed to aspergillus conidia and devoid of alloreactivity, that were employed to treat IA in 10 recipients of haplo-HSCT (35). Emergence of circulating pathogen-specific T cells were associated with control of Aspergillus antigenemia and infectious mortality.

### Pathogen-Specific T Cells: Preliminary Clinical Results for PyVs and HHV6

Cell therapy has been employed also for the treatment of other infections, such as polyomaviruses and HHV6. Although very preliminary, initial experiences with BKPyV-specific cells are promising (36, 69), as 13 of 14 patients treated for BKV-associated hemorrhagic cystitis within a clinical trial of third-party banked multivirus-specific T cell therapy in allogeneic HSCT experienced complete resolution of gross hematuria within 1–2 months (36). Of the two patients treated for virus-related nephropathy, one responded to treatment by ameliorating renal function. In 50% of the treated patients, an increase in BKPyV-specific immune response was observed. The main side effects were recurrence or new onset of GVHD in 16% of the whole study cohort and transient hydronephrosis and a decrease in renal function in one patient who received VSTs as treatment for BKPyV HC. The latter, associated with a concomitant bacterial urinary tract infection, could have also been due to lysis of infected cells in renal tubular cells.

Four patients, reported in two studies, were treated for JCPyV PML (14, 74). One pediatric HSCT recipient received donor JCPyV-specific T cells, that was associated with reconstitution of specific viral immunity, clearance of viral DNA from the cerebrospinal fluid (CSF) and disease control with remarkable neurological improvement, in the absence of immune reconstitution syndrome (14). Three patients were treated with third-party allogeneic BKPyV-specific T cells, based on reported observations of a certain degree of cross-reactivity between PyV BK and JC due to high homology (74). The CBT recipient fully recovered. In the other two patients, viral load was cleared or reduced in CSF, with the patients showing neurologic improvement with residual deficit in one case, and disease progression in the other. Two of the patients had immune reconstitution syndrome. Phase I or I/II trials are currently underway.

Two patients with HHV6 infections were treated with T cells specific for U11, U14, and U90 within a clinical trial of third-party banked multivirus-specific T cell therapy in allogeneic HSCT (36). One patient was treated for HHV6 encephalitis and the other for HHV6 viremia with fevers and symptoms of bone marrow suppression, including neutropenia. Both patients showed decreased viral load and normalization of clinical disease.

### Experience With Multivirus-Specific T Cells

Most of the cell therapy experience regards treatment of CMV and EBV infections. However, patients with multiple infections have a worse outcome (19), and in the pediatric population or in recipients of haplo-HSCT, the impact of other viral infections, such as adenovirus or HHV6, has important implications for overall survival (8, 87). Thus, the possibility to produce in a single process VSTs specific for multiple viruses is crucial for progress in the field. Proof of principle studies have been conducted, that demonstrated feasibility and preliminary efficacy of controlling viral reactivation after allogeneic HSCT by multivirus-specific VST of HSCT donor or third-party origin, obtained by *ex-vivo* stimulation with virus-transduced EBV lymphoblastoid cell lines (75–77, 82, 84), dendritic cells nucleofected with plasmids encoding for viral proteins or pulsed with viral peptides (78, 79, 81), or directly with 15-mer peptide pools from immunogenic viral proteins (36, 80) (Table 2).

Prophylactic or curative administration in a total of 82 patients treated with HSCT donor-derived cells and 96 third-party donor cells showed responses in the range of 80–95 and 70–100%, respectively (Table 2). Clinical benefit could be demonstrated also in patients treated for multiple coincident infections (36). Although clinical responses have been registered for all targeted viruses, evidence of T cell expansion in the peripheral blood of treated patients is mainly seen for viruses with large memory cell pools, such as CMV and EBV, while, due to the small size of their memory compartment, immune responses to AdV or HHV6 do not seem to be boosted unless a reactivation is underway. Indeed, antigenic competition that will ensue when engaging multiple target antigens within the same culture, will determine a preferential expansion of T cells recognizing the immunodominant specificities of viruses with large memory cell pools. This will impact on the composition of multivirus-specific T cell products, as T lymphocytes directed to certain non-immunodominant targets, as well as to viruses with low-frequency memory T cells, will be underrepresented, and it may also ultimately impact on efficacy.

## Current Limitations of T Cell Therapy for Infections

There are several hurdles that concur in limiting the use and the clinical efficacy of pathogen-targeted T cell therapy. First of all, production of pathogen-targeted T cells have been so far mostly confined to a relatively small numbers of academic centers with required Good Manufacturing Practice (GMP) expertise and facilities, that have limited ability to provide widespread access to these therapies. Moreover, for some patient categories, such as recipients of HSCT from pathogen-naïve donors, expansion of dedicated T cell products may not be feasible.

In addition, the appropriate timing and schedule for T cell delivery, as well as T cell dose or optimal cell product composition, have not been yet established, due to the presence of many different confounding variables, such as transplant ad infectious disease setting, use of *in vitro* or *in vivo* T-cell depletion, and immunosuppressive regimens. These issues will have to be addressed in future controlled comparative trials.

Finally, HSCT recipients treated with steroids or calcineurin inhibitors (CI) for GVHD are among those at highest risk of infectious complications. However, in these patients cell therapy has the least chance of success, as steroids have a direct cytopathic effect, and CI impair T cell expansion potential. Recently, preclinical studies have demonstrated the feasibility of producing pathogen-specific single or multivirus-specific T cells resistant to steroids (88), or to CI (89, 90), by genetic modification, and clinical studies are underway to assess safety and preliminary efficacy.

## Improving Access to Cell Therapy

### Manufacturing VST From Antigen-Seronegative Donors

Pediatric recipients who reactivate viral infections after HSCT from virus-naïve stem cell donors are at high risk of developing complications. It has been shown that it is possible to prime tumor- or virus-specific responses by delivering viral antigens presented by professional antigen-presenting cells in the presence of activating/homeostatic cytokines (91, 92). Stimulation by dendritic cells pulsed with EBV LCL, or stimulation with EBV-LCL, either with subsequent selection of CD25-positive T-cells, or in the presence of cytokines, such as IL-7 and/or IL-12, have all been described (92–94). The latter approach was demonstrated effective when employed to expand EBV-CTL that were successfully infused *in vivo* to treat a disseminated PTLD, unresponsive to multiple courses of rituximab and chemotherapy, in a pediatric recipient of unrelated HSCT from a EBV-seronegative donor (95).

Multivirus (CMV, EBV, and adenovirus)-specific T-cells have been activated and expanded from CB, by stimulation with DC or LCL pulsed with a CMV-pp65 overlapping peptide library, in the presence of IL-7, IL-12, and IL-15. The primed cells were only able to recognize atypical pp65 epitopes, but when administered to CBT recipients mediated CMV-directed activity in one patient experiencing viral reactivation (82).

### Third-Party Banked VST

Donor-derived VST infusions are not always feasible in clinical practice, due to impossibility to obtain starting material from the donor, as in UD or CB transplantation. Moreover, rapid disease progression may not allow the time required for dedicated production. A practical approach to overcome these issues is to employ banked, HLA-typed VST obtained from healthy donors, selected for a candidate recipients on the basis of the most closely matched line with specific activity against a given pathogen through one or more shared HLA epitopes.

Theoretically, third-party VST could have short persistence *in vivo*, with limited clinical benefit, as the partial HLA disparity may induce allorecognition by recipient T cells. Alloresponses by infused third-party cells may, in turn, cause GVHD. So far, results have been encouraging, with only one report of bystander-induced liver GVHD (96). Seminal data were obtained in solid organ-transplanted patients with EBV PTLD: the response rate in the 33 patients enrolled in the first phase II trial, that included 6 HSCT recipients, was 52% at 6 months (70). Since then, third-party VST have been effectively used also in the setting of HSCT (36, 62, 71–73, 83, 84, 96), demonstrating that the approach is feasible without inducing a higher rate of GVHD than HSCT donor-derived VST, while producing significant clinical responses. A recent study demonstrated safety and efficacy of third-party rapidly-generated single-culture donor VST that recognized 12 antigens from EBV, AdV, CMV, BKPyV, and HHV6 in 38 patients enrolled in a phase II trial. Importantly, clinical benefit could be demonstrated also in seven patients treated for multiple coincident infections (36). VST banks recently created include products characterized for epitope specificity and HLA restriction elements, to further refine selection of the best VST for each patient.

## Concluding Remarks

It is likely that VSTs will have an increasing role as therapeutics in the prevention and management of viral infections after HSCT, due to high rate of response and limited toxicity profile observed in reported studies. However, many issues are still open, and will need to be addressed in future studies, such as the most suitable predictive markers for response, the identification of patients at risk of treatment failure, optimal treatment schedule in different clinical settings, and choosing adequate end-point for future clinical trials.

The majority of subjects treated to date with cell therapy for infections have received dedicated donor T cells, but this approach may not be best suited for widespread cost-effective access, since these are personalized medicines that are produced on-demand through a complex and costly supply chain. The development of new methodologies to obtain rapid manufacture of third-party T cells, refinement of strategies to allow adequate selection of the “best VST” for each candidate patient and the possibility to widen applicability to setting beyond HSCT, has prompted considerable interest from the industry to bring to the market third-party cellular therapies. This process will allow to benefit patients through better T cell therapy access.

## Author Contributions

SBa, FC, PZ, GG, SBo, EB, JB, MS, CDF, ML, MZ, and PC all participated in writing the manuscript. SBa, MZ, and PC co-edited the final version of the manuscript. All of the authors have read and approved the final manuscript.

## Conflict of Interest

The authors declare that the research was conducted in the absence of any commercial or financial relationships that could be construed as a potential conflict of interest.

## Abbreviations

allo-HSCT: allogeneic hematopoietic stem cell transplantation
AdV: adenovirus
BKPyV: polyomavirus BK
JCPyV: polyomavirus JC
CMV: Cytomegalovirus
HHV6: human herpes virus 6
EBV: Epstein-Barr virus
PTLD: Post-transplant lymphoproliferative disease
HC: hemorrhagic cystitis
PML: progressive multifocal leukoencephalopathy
UD: unrelated donor
NRM: non-relapse mortality
DLI: donor lymphocyte infusions
CTL: Cytotoxic T lymphocytes
a/cGVHD: acute/chronic graft vs. host disease
VSTs: virus-specific T cells
CI: calcineurin inhibitors.
